# Belgian Cross-Sectional Epidemiological Study on Zoonotic Avian *Chlamydia* spp. in Chickens

**DOI:** 10.3390/microorganisms12010193

**Published:** 2024-01-18

**Authors:** Anne De Meyst, Pieter De Clercq, Jelmer Porrez, Tom Geens, Lutgart Braeckman, Sander Ouburg, Servaas A. Morré, Daisy Vanrompay

**Affiliations:** 1 Department of Animal Sciences and Aquatic Ecology, Faculty of Bioscience Engineering, Ghent University, 9000 Ghent, Belgium; anne.demeyst@ugent.be (A.D.M.); pieterdc.declercq@ugent.be (P.D.C.); jelmer.porrez@irc.vib-ugent.be (J.P.); 2 Research and Analytics, Liantis, 8200 Bruges, Belgium; tom.geens@liantis.be; 3 Department of Public Health and Primary Care, Faculty of Medicine and Health Sciences, Ghent University, 9000 Ghent, Belgium; lutgart.braeckman@ugent.be; 4 Research & Development, Microbe&Lab BV, 1105 AG Amsterdam, The Netherlands; s.ouburg@microbenlab.com (S.O.); samorretravel@yahoo.co.uk (S.A.M.); 5 Department of Genetics and Cell Biology, GROW School for Oncology and Reproduction, Maastricht University, 6229 ER Maastricht, The Netherlands; 6 Dutch *Chlamydia trachomatis* Reference Laboratory, Department of Medical Microbiology, Faculty of Health, Medicine & Life Sciences, Maastricht University, 6229 ER Maastricht, The Netherlands; 7 Department of Molecular and Cellular Engineering, Jacob Institute of Biotechnology and Bioengineering, Sam Higginbottom University of Agriculture, Technology and Sciences, Allahabad 211007, Uttar Pradesh, India

**Keywords:** *Chlamydia*, chicken, zoonosis, psittacosis, *Chlamydia psittaci*, *Chlamydia gallinacea*, *Chlamydia abortus*, backyard, poultry industry

## Abstract

*Chlamydia psittaci*, *Chlamydia gallinacea,* and *Chlamydia abortus* are the most common *Chlamydia* spp. in chickens and have a confirmed or suggested zoonotic potential. No recent data are available on their prevalence and impact in the Belgian chicken industry or in the recreational chicken branch. Therefore, a cross-sectional epidemiological study was executed where samples were collected from both factory-farmed and backyard chickens. More specifically, pharyngeal chicken swabs were obtained from 20 chicken farms, 5 chicken abattoirs, and 38 different backyard locations and were analyzed using species-specific Polymerase Chain Reactions (PCRs) for the presence of the three avian *Chlamydia* spp. To investigate their zoonotic potential, samples were simultaneously collected from 54 backyard chicken caretakes and 37 professional chicken caretakers or abattoir employees and analyzed using species-specific PCRs as well. This study confirmed the presence of DNA of all three *Chlamydia* species in both the chicken industry and backyard settings. *Chlamydia psittaci* was the most prevalent in the industry chickens (11.0%), whereas *Chlamydia gallinacea* was the dominant species in the backyard chickens (14.5%). *Chlamydia abortus* infections were more common in the commercial chickens (9.0%) compared to the backyard chickens (2.6%). The DNA of all three species was also detected in humans (3.9% *Chlamydia psittaci*, 2.9% *Chlamydia gallinacea,* and 1.0% *Chlamydia abortus*).

## 1. Introduction

Since 2008, the chicken industry in Belgium has progressively gained significance. From 20.1 million broiler chickens and 11.5 million laying hens in 2008, the chicken herd in Belgium nearly doubled to 36.9 million broilers and 16.2 million laying hens in 2021 [1]. In industrial poultry farming, animals are typically raised at high densities in confined spaces, which creates a favorable environment for the propagation and spread of infectious diseases. Despite the implementation of intensive husbandry practices, these chickens are vulnerable to infections [2]. The trend of keeping backyard chickens and raising chickens as pets is also on the rise. This growth can be attributed to an increasing interest in humane and organic animal products and sustainable agriculture with a farm-to-table food supply [3]. Biosecurity measures and health management protocols are often lacking in backyards. Since these animals regularly come into contact with wild birds, which represent a potential reservoir for infectious diseases, these chickens are also vulnerable to infections [4].

*Chlamydia* (*C.*) infections are regularly reported in chickens. These obligate intracellular bacteria have a biphasic life cycle and are classified into 14 recognized species [5]. Among these, *C. psittaci*, *C. gallinacea*, *C. abortus*, *C. muridarum*, *C. suis*, and *C. pecorum* are able to infect poultry. The first three species naturally infect poultry, whereas the latter three are only sporadically detected in birds, presumably after close contact of the birds with their respective natural hosts [6,7].

*C. psittaci* is the oldest known *Chlamydia* agent that infects birds. This airborne bacterium has been isolated from over 460 free-living or pet bird species, including domestic poultry such as turkeys, chickens, and ducks. *C. psittaci* can be classified into nine genotypes (A–F and E/B in avian species and WC and M56 in mammals), along with several provisional genotypes (1V, 6N, Mat116, R54, YP84, CPX0308, I, and J) isolated from psittacine and wild birds, which currently lack detailed characterization [8,9,10]. Of the seven well-characterized avian genotypes, each exhibits a distinct host preference, with genotypes A, B, C, D, and E/B having already been identified in chickens [11,12]. *C. psittaci* is an airborne pathogen but horizontal trans-shell transmission is also considered possible [13]. An infection in birds can cause respiratory disease accompanied by symptoms such as respiratory distress, nasal and ocular discharge, weight loss, reduced egg production, and lethargy [14]. In 2013, 19 Belgian chicken farms were tested for the presence of *C. psittaci*, and the agent was detected in 18 farms using both culturing and PCR [2]. This high prevalence contradicts reports from other countries where *C. psittaci* has been hardly detected in chickens [15,16,17]. *C. psittaci* can also be transmitted to humans, resulting in a disease called psittacosis, and can be characterized as a flu-like illness with pneumonia, fever, and headache as the primary symptoms. As *C. psittaci* is transmitted mostly through the inhalation of contaminated excretions, individuals that come into close contact with birds are an important risk population [11,18]. Transmission from chickens to chicken industry workers has been extensively investigated in Belgium and appears to regularly occur [2,19,20].

*C. gallinacea* is a recently discovered species which has been isolated from both poultry [21] and parrots [22]. Its pathogenicity is not well understood because previous studies only reported a minor reduction in weight gain in broiler chickens [7,23]. Further research is needed to fully assess the impact of this pathogen on avian health. Since its discovery, *C. gallinacea* has been frequently detected and is nowadays considered endemic in chickens. In 2016 and 2018, respectively, *C. gallinacea* DNA was detected in 81.2% of *Chlamydia*-positive chickens in China and in 47.0% of layer farms in the Netherlands [7,16]. Nevertheless, *C. gallinacea*’s presence has not been examined in Belgium yet. Unlike *C. psittaci*, *C. gallinacea* is transmitted via the fecal–oral route. Vertical transmission via eggshell penetration is also considered possible [24]. Marchino et al. [5] were the first to report the presence of *C. gallinacea* DNA in human sputum samples, originating from poultry workers that were exposed to *C. gallinacea*-positive chickens. The presence of *C. gallinacea* DNA in both the poultry workers and the chickens indicated possible bird-to-human transmission, though the exact mode of transmission remains uncertain. None of these employees showed signs of respiratory illness.

*C. abortus* is the etiological agent of the enzootic abortion of ewes [25]. Next to its presence in small ruminants, it has been reported in poultry and wild birds [8,10,26,27]. As these avian *C. abortus* strains are closely related to *C. psittaci*, they were called *C. psittaci*/*C. abortus* intermediates or atypical *C. psittaci*. However, intensive genomic research led to the decision to expand the species of *C. abortus* to not only include a mammalian subtype but also an avian subtype [28,29,30]. As of yet, these novel avian *C. abortus* strains have not been associated with any disease, and their zoonotic potential remains unconfirmed. However, this is highly likely, as mammalian *C. abortus* strains are known zoonotic agents which cause flu-like illness or even abortion in pregnant women [31,32].

In the past decade, our understanding of avian chlamydia has significantly expanded, with the identification of new species and subspecies. However, there is currently a lack of epidemiological data on avian *Chlamydia* spp. in Belgium. Recognizing the growing significance of chickens as both pets and food sources, the prevalence of both known and newly discovered chlamydia strains was examined in commercial and domestic chickens. Additionally, considering the confirmed or suspected zoonotic potential of these species, our study aimed to investigate the presence of these *Chlamydia* spp. in humans.

## 2. Materials and Methods

### 2.1. Sampling

#### 2.1.1. Sampling in the Chicken Industry

The chicken samples were collected from Belgian abattoir and farm chickens, with the help of volunteering veterinarians. More specifically, pharyngeal swabs were collected from 20 chicken farms (10 chickens per farm or flock) and 5 chicken abattoir flocks (40 chickens per flock). Among these, 8 farms were situated in Flanders and 12 in Wallonia, whereas all abattoirs were located in Flanders. The 400 pharyngeal swabs were collected individually by rubbing the palate cleft and upper pharyngeal papillae of the chickens with an aluminum rayon-tipped swab (Copan, Brescia, Italy). The swabs were put into 2 mL DNA stabilization reagent (Roche, Basel, Switzerland) and stored at 4 °C. After a maximum of 24 h, they were transported to the laboratory, where they were shaken for 1 h at 4 °C and stored at −80 °C until further processing.

The human samples were collected from volunteers with an occupational exposure to chickens. A total of 15 chicken farm employees and 33 abattoir workers cooperated in the study. Following informed consent, the employees were provided with a sampling package containing a FLOQSwab^®^ (Copan), storage buffer, a questionnaire, and a detailed manual. The volunteers were asked to complete the questionnaire and to collect a pharyngeal swab by rubbing the swab near their tonsils. Similar to the chicken samples, the swab was put into DNA stabilization reagent, and the packages were stored at 4 °C until transport to the laboratory within a maximum of 24 h. All samples were pseudonymized, shaken for 1 h at 4 °C, and stored at −80 °C until further processing.

It is worth noting that despite reaching out to numerous Belgian chicken farms and abattoirs, only a small percentage agreed to cooperate.

#### 2.1.2. Sampling in Backyards

Pharyngeal swabs were also collected from domestic chickens, located in 38 different backyards over Flanders (2 chickens per backyard or flock). From these 38 different households, 54 domestic chicken owners volunteered to provide samples as well. These volunteers were recruited through Belgian associations of yard animals and social media platforms. Participation was limited to individuals who owned at least two backyard chickens, with a maximum of two chicken caretakers allowed to participate per household. After informed consent, a sampling package was sent per post to the households, containing a detailed manual, a questionnaire, the necessary material to collect a pharyngeal swab per volunteer, and pharyngeal swabs from two backyard chickens individually (see details above). After the sample collection by the volunteers, the samples were stored at 4 °C, and within 24 h, the package was sent by express post to the laboratory. All samples were pseudonymized, shaken for 1 h at 4 °C, and stored at −80° until further processing.

Similar to the industrial sampling process, despite reaching out to a substantial number of individuals, only a few people responded.

### 2.2. Molecular Analysis

The DNA was extracted from all pharyngeal swabs stored in DNA stabilization reagent. Therefore, the QIAamp^®^ DNA mini kit (QIAGEN, Antwerp, Belgium) was used according to the manufacturer’s guidelines for the DNA extraction from buccal swabs. All the DNA samples were subjected to three species-specific PCRs.

#### 2.2.1. *C. gallinacea*-Specific Real-Time PCR

The presence of *C. gallinacea* DNA was examined using a real-time PCR developed by Laroucau et al. [33] and further described by Heijne et al. [16]. This PCR detects the *enoA* gene with a sensitivity of 5 copies/reaction, using the primer sequences FW “CAATGGCCTACAATTCCAAGAGT” and REV “CATGCGTACAGCTTCCGTAAAC” and probe sequence “FAM-ATTCGCCCTACGGGAGCCCCTT-TAMRA”. Each reaction consisted of 5 µL of the DNA template, 10 µL of TaqMan™Fast Universal PCR Master Mix (2×) (Applied Biosystems, Dublin, Ireland), 1 µM of the forward and reverse primers, 0.2 µM of the probe, and 1 unit of AmpErase™ Uracil N-glycosylase (Applied Biosystems). DNA amplification was performed with the following cycling conditions: 37 °C for 5 min, 95 °C for 20 s, followed by 50 cycles of 95 °C for 3 s and 60 °C for 30 s. According to validation with an in-house control plasmid, samples with a Ct value lower than 38 were considered positive.

#### 2.2.2. *C. abortus*-Specific Real-Time PCR

The presence of *C. abortus* DNA was examined using a commercial real-time PCR (Path-*C. abortus*, PrimerDesign Ltd., Liverpool, UK). This kit exclusively detects the 3-deoxy-D-manno-2-octulosonic acid transferase (*kdtA*) gene of both mammalian and avian *C. abortus* strains with a sensitivity of 10 copies/reaction. The primer and probe sequences are kept confidential by the company. Each reaction consisted of 10 µL oasig™ Lyophilized 2× Master Mix (PrimerDesign Ltd.), 1 µL of the primer/probe mix, 4 µL of nuclease-free water, and 5 µL of the DNA template. DNA amplification was performed with the following cycling conditions: 95 °C for 2 min, followed by 50 cycles of 95 °C for 10 s and 60 °C for 60 s. According to validation with a control plasmid (included in the kit), samples with a Ct value lower than 40 were considered positive [34].

#### 2.2.3. *C. psittaci*-Specific Nested PCR

The presence of *C. psittaci* DNA was examined using a nested PCR developed by Van Loock et al. [35]. This PCR detects all genotypes with a sensitivity of 1 IFU, using the following primer sequences: FW_extern “CCTGTAGGGAACCCAGCTGAA”, REV_extern “GGTTGAGCAATGCGGATAGTAT”, FW_intern “GCAGGATACTACGGAGA”, REV_intern “GGAACTCAGCTCCTAAAG”. Next to 1.25 µM of each external primer, a first-round reaction consisted of 50 mM of KCl (Sigma-Aldrich, Hoeilaart, Belgium), 20 mM of Tris-HCl (Sigma-Aldrich), 3.5 mM of MgCl_2_ (Sigma-Aldrich), 0.1% of Tween20 (Sigma-Aldrich), 200 µM of each dNTP (Invitrogen, Carlsbad, CA, USA), 1.25 units of DreamTaq DNA polymerase (Thermo Scientific, Illkrich, France), and 5 µL of the DNA template. The second round of PCR was executed under similar conditions but with 10 µM of each internal primer per reaction. The cycling conditions for the first round were as follows: 95 °C for 5 min, followed by 20 cycles of 95 °C for 1 min, 59 °C for 2 min, and 72 °C for 3 min, followed by 72 °C for 5 min. The second round of PCR used the same conditions but with 25 cycles of 95 °C for 1 min, 47 °C for 2 min, and 72 °C for 3 min [35].

#### 2.2.4. *C. psittaci* Genotyping

The *C. psittaci*-positive human samples were submitted to genotyping according to a genotype-specific real-time PCR developed by Geens et al. [36]. This PCR detects genotypes A–F and E/B with a sensitivity of 10 copies/µL DNA extract.

### 2.3. Statistical Analysis

During the sampling process at the farms and abattoirs, data regarding the flock size, the presence of other farm animals, the age of the sampled chickens, and the chicken type were recorded. In order to determine which risk factors were associated with *Chlamydia*, *C. psittaci*, *C. gallinacea*, and *C. abortus* infections in the factory-farmed chickens, a generalized linear mixed model was implemented with a binary logistic response. A manual forward stepwise approach was applied, with the farm or abattoir included as a random effect in each iteration. A likelihood ratio test was conducted to determine which variables significantly contributed to the model. Additionally, the volunteers that participated in the study completed questionnaires informing us on their general health status, (non)professional activities, as well as their contact frequency with animals. Correlations between these risk factors and the *Chlamydia*, *C. psittaci*, *C. gallinacea*, and *C. abortus* positivity of the human pharyngeal swabs were determined using Fisher’s exact test, as this test allows for small sample sizes. The examined risk factors included gender, age, respiratory symptoms at the moment of sampling, respiratory diseases at the moment of sampling, and exposure to poultry or other animals. For volunteers who also owned backyard chickens, additional factors such as the origin of the chickens, any symptoms exhibited by the chickens (at the moment of sampling or before), the presence of other animals, and whether the chickens tested positive for *Chlamydia*, *C. psittaci*, *C. gallinacea*, or *C. abortus* were also investigated. The information from the questionnaires was also used to analyze correlations between *Chlamydia* infections in backyard chickens and the origin of the chickens, current or previous symptoms noticed by the owner, and the presence of other animals in the backyard. Again, Fisher’s exact test was employed as it allows for smaller sample sizes. All statistical analyses were performed in SPSS Statistics 28 with a significance level of 0.05.

## 3. Results

### 3.1. Analysis of Chicken Samples

Table 1 provides an overview of the molecular analysis conducted on the pharyngeal swabs of the commercial chickens. After eliminating the double positives that arose from mixed infections, 93 out of 400 chickens tested positive for *Chlamydia* (23.3%). Most of the *Chlamydia*-positive chickens were infected with *C. psittaci* (44/93; 47.3%), followed by *C. abortus* (36/93; 38.7%) and *C. gallinacea* (32/93; 34.4%). The prevalence rates in the farm chickens were higher compared to rates in the abattoir chickens (overall *Chlamydia* positivity of 33.0% compared to 13.5%). Mixed infections with two or three different species were regularly detected. Both *C. psittaci* and *C. gallinacea* were equally present in the 25 tested flocks (16/25 flocks; 64.0%). *C. abortus* DNA was detected in 14/25 flocks (56.0%), resulting in an overall *Chlamydia* flock positivity of 23/25 (92.0%). Only two tested flocks were completely *Chlamydia*-negative: location 3 holding broilers and location 4 holding slow-growing broilers.

A risk factor analysis was conducted using a generalized linear mixed model. None of the tested factors (chicken type, age, flock size, or presence of other animals) seemed to significantly contribute to the model. 

PCR was also performed on pharyngeal swabs obtained from 76 backyard chickens at 38 different locations. The results of the molecular analysis can be found in Table 2. From the 76 backyard chickens, 17.1% were *Chlamydia*-positive. Whereas *C. psittaci* and *C. abortus* were only found in, respectively, 1 and 2 birds, *C. gallinacea* was present in 14.5% (11/76) of the birds. Only one bird had a mixed infection (*C. abortus* and *C. gallinacea*).

The results of Fisher’s exact tests demonstrated a significant association between the prevalence of *C. abortus* in the chickens and the clinical signs observed by the owners before sampling (*p* = 0.015). These symptoms included pumping breath, open beak breathing, abnormal breathing sounds, and diarrhea.

### 3.2. Analysis of Human Samples

All the human pharyngeal swabs were submitted to three PCRs to detect *C. psittaci*, *C. abortus*, and *C. gallinacea* DNA. The results of these analyses can be found in Table 3. Four people were found positive for *C. psittaci* (3.9%), three people for *C. gallinacea* (2.9%), and one for *C. abortus* (1.0%). One person had a mixed *C. gallinacea*/*C. psittaci* infection. Of these seven people, six only had recreational contact with chickens. The remaining *Chlamydia*-positive person was an abattoir worker with frequent occupational exposure to *Chlamydia*-infected chickens.

Interestingly, Fisher’s exact tests indicated a correlation between the presence of *Chlamydia* DNA in humans and *Chlamydia* infections in chickens (*p* = 0.028). Additionally, there was a significant correlation between human *C. gallinacea* positivity and *C. gallinacea* infections in chickens, as well as between human *C. abortus* positivity and *C. abortus* infections in chickens, with respective *p*-values of 0.017 and 0.019. *C. psittaci* detection in human samples and animal samples was not correlated (*p* = 0.889). However, it must be mentioned that multiple chickens of the same household were infected with *C. gallinacea* or *C. abortus*, possibly influencing the statistical test.

The descriptives of the seven positive humans are presented in Table 4. For each person that was infected with *C. gallinacea* or *C. abortus*, the species was also found in the samples of their chickens. However, for *C. psittaci*, there was no link between human infection and animal infection, as indicated in the statistical results. Therefore, the *C. psittaci*-positive human samples were submitted to genotyping with a genotype-specific real-time PCR. In all four samples, genotype D was detected with a Ct value of 38.6 (human 3), 39.2 (human 4), 37.9 (human 5), and 38.3 (human 6). Several people reported clinical symptoms at the time of sampling, which can be linked with *Chlamydia* infection. The Ct values of the *C. abortus* and *C. gallinacea*-positive samples were rather high, indicating a low excretion rate (average Ct value of 37.7).

## 4. Discussion

Over the past years, the knowledge on avian chlamydia has evolved tremendously. Continuous epidemiological research is needed to provide new insights into the prevalence, transmission, and risk factors associated with infection. Therefore, this study examined its potential impact in both the professional and recreational chicken branch.

In the first phase of this study, pharyngeal swabs were collected from 400 commercial chickens, sourced from farms and abattoirs. These samples were subjected to PCR analysis. The overall *Chlamydia* positivity rate in the commercial chickens was 23.3%. *C. psittaci* accounted for the majority of infections (47.3%), followed by *C. abortus* (38.7%) and *C. gallinacea* (34.4%). Sukon et al. [37] determined the global *Chlamydia* prevalence in Galliformes to be around 32.0% (95% CI 20.6%—46.1), combining the results of 15 different studies in a systematic review and meta-analysis. The mean infection rate found in this study (23.3%) is lower than the global average but still located within the 95% CI.

Most of the commercial chicken flocks were infected with *C. psittaci* (16/25; 64.0%) and *C. gallinacea* (16/25; 64.0%), followed closely by *C. abortus* (14/25; 56.0%). These prevalence rates differ from those reported in other studies, which predominantly found *C. gallinacea* in chickens [7,16]. For instance, in Mexico, *C. gallinacea* was found in 7.1% of controlled environment commercial farms, while *C. psittaci* was not detected [15]. In another study from Germany, which investigated the prevalence of *C. gallinacea* and *C. psittaci* in poultry slaughterhouses, *C. gallinacea* was detected in 48.5% of *Chlamydia*-positive poultry flocks, but *C. psittaci* was not found [38]. In contrast, Belgian studies conducted in 2013 and 2014 (before the discovery of avian *C. abortus* and *C. gallinacea*) reported remarkably high prevalence rates of *C. psittaci* in chicken flocks. Specifically, Yin et al. (2012) determined the flock seropositivity to be between 90.0 and 96.0% using a *C. psittaci* MOMP-based antibody ELISA, while Lagae et al. (2014) found approximately 95.0% flock positivity using a PCR assay developed by Van Loock et al. [2,12,35]. The precise reasons behind these discrepancies in prevalence rates remain unclear. One possibility is that the ELISA employed by Yin et al. (2012) was less specific and cross-reacted with other species. However, this is not the case for the PCR used in both this study and the one conducted by Lagae et al. (2014). Another possible explanation could be a difference in antibiotics administration. In recent years, the Belgian livestock sector has made many efforts to reduce antibiotics usage, and the administration of preventive antibiotics has even been prohibited since January 2022 (Regulation EU 2019/6) [39]. However, as the studies in question did not report antibiotic usage, it remains uncertain whether this factor could account for the observed differences. A last possibility is that the prevalence of *C. psittaci* has decreased over time, while *C. abortus* and *C. gallinacea* are emerging in commercial chickens in Belgium. The first report on *C. abortus* in chickens was only published in 2017, where a prevalence rate of 15.4% was found when testing 182 healthy poultry flocks. As *C. abortus* was detected in 56.0% of the tested chicken flocks in this study, avian *C. abortus* strains can be considered widespread among chickens.

A risk factor analysis determined that the chicken type, age, flock size, or presence of other animals did not correlate with the *Chlamydia* infection rate. However, earlier studies indicated that some of these factors can influence the prevalence rates. For instance, Heijne and colleagues (2018) found that the age of hens was significantly correlated with the *C. gallinacea* infection rate, with a peak around 40–60 weeks. Next to hen age, the use of bedding and the presence of horses were also significantly correlated with the *C. gallinacea* infection rate [16]. On the other hand, it is known that turkeys experience a *C. psittaci* infection wave around 3–6 weeks of age when maternal antibody titers have declined [40]. This is presumably also the case for chickens. Additionally, *Chlamydiaceae* positivity has been associated with, among other things, the type of farming (egg, meat, or mixed production); presence of other *Chlamydiaceae* on the farm; presence of free-range sheds, grass, or bushes; removal of dead animals at the end of the cycle; and high stocking densities [2,5].

In the second phase of this study, pharyngeal samples were collected from 76 backyard chickens from 38 backyards and analyzed using PCR. *C. gallinacea* was the predominant agent, found at 23.7% of the tested locations. *C. psittaci* and *C. abortus* were only detected at one location each (2.6%). The lower infection rate of *C. psittaci* can perhaps be explained by its mode of transmission. This pathogen is transmitted via aerosols whereas *C. gallinacea* is transmitted via the fecal–oral route [14,24]. The latter transmission route is more likely to occur in open-air backyards with poor biosecurity measures and contact with possibly infected wild birds. In an industrial setting, chickens are housed in confined spaces, allowing an easier transfer of airborne pathogens [3,4]. Mammalian *C. abortus* strains are transmitted orally but transmission via the inhalation of aerosols has also been suggested [25,31]. Whether this is also the case for avian *C. abortus* strains is not known, but it would explain the lower prevalence in backyard chickens, even though the species is known to occur in wild birds [10]. Domestic chicken owners indicated that none of the infected chickens showed any clinical signs. This is in concordance with other studies which describe limited/no pathogenicity of the *Chlamydia* strains circulating in chickens [8,23,37]. To our knowledge, this is the first study where samples were collected from domestic chickens and not from backyard farms. The prevalence rates are therefore difficult to compare with other studies. A study from Mexico in backyard farms reported a *Chlamydia* farm positivity of 75.0% (12/16), with *C. gallinacea* as the only detected agent [15]. In backyard farms in Italy, 15.0% of the sampled chickens were *Chlamydia*-positive, with *C. gallinacea* found at 9/16 farms [17]. These higher prevalence rates are not surprising, as backyard farms harbor more animals, allowing for more transmission events to occur.

Statistical analysis revealed a significant correlation between the *C. abortus* infections in backyard chickens and formerly observed clinical symptoms in these chickens. The reported clinical signs are characteristic of a *C. psittaci* infection: pumping breath, open beak breathing, abnormal breathing sounds, and diarrhea. However, it remains uncertain whether these clinical signs were a consequence of a *C. abortus*, *C. psittaci*, or *C. gallinacea* infection or an infection with another respiratory pathogen. It is plausible that *C. abortus* infection could lead to respiratory symptoms, given its close relation to *C. psittaci*, but until today, *C. abortus* has only been detected in asymptomatic carriers [8,29].

With the One Health approach in mind, this study also aimed to investigate the zoonotic potential of these three *Chlamydia* agents. Therefore, pharyngeal swabs were collected from both occupational and recreational chicken caretakers and examined using PCR. Of the 48 pharyngeal swabs collected from occupational chicken caretakers, only one sample was *Chlamydia*-positive (*C. psittaci* genotype D). That only 1 professional chicken caretaker tested positive for *Chlamydia* is in strong contrast with an earlier Belgian study, reporting the transmission of *C. psittaci* to 93.5% of the farmers [2]. However, it is concordant with other studies reporting that transmission from *Galliformes* is rather rare due to the limited virulence of these strains [11,37]. Pharyngeal swabs were also collected from 54 volunteers who owned backyard chickens. DNA of all three *Chlamydia* spp. was found in these samples with a prevalence of, respectively, 5.6%, 5.6%, and 1.9% for *C. psittaci*, *C. gallinacea*, and *C. abortus*. This is the first report where *C. gallinacea* DNA and *C. abortus* DNA were detected in human pharyngeal swabs. *C. psittaci* was detected in three individuals who owned *C. psittaci*-negative animals, but genotype D typing suggests that chickens were the probable infection source. The four individuals that tested positive for *C. gallinacea* or *C. abortus* owned chickens that also tested positive for *C. gallinacea* or *C. abortus*, indicating possible transmission from the chickens to the owners. The correlation between the infected backyard chickens and the presence of *C. gallinacea* or *C. abortus* in the human swabs was even found to be significant according to Fisher’s exact test. However, the high Ct values (average Ct value of 37.69) suggest that contamination during sample collection cannot be ruled out as a possible explanation for these findings. The risk of contamination during the sample processing and analysis was minimized by analyzing the human and animal samples separately, thus ensuring that cross-contamination did not occur in the lab. However, we were not able to further confirm the transmission, as culture and sequencing attempts failed due to the low excretion loads. Three people who tested positive for *C. psittaci* and/or *C. gallinacea* suffered from clinical signs, including headache, stomachache, dry skin, dry eyes, and rhinitis. However, these symptoms were not found to be significantly correlated with infection. One person was infected with SARS-CoV-2 at the time of sampling and was experiencing respiratory symptoms likely attributed to this viral infection. Nevertheless, it remains uncertain whether chlamydial infection contributed to the respiratory complaints or not. Psittacosis can be accompanied by respiratory signs, particularly when infected with *C. psittaci* genotypes A, B, or E/B [11,33,41]. On the other hand, *C. psittaci* genotype D has been previously detected in poultry workers who exhibited respiratory symptoms [2]. *C. gallinacea* has not been associated with disease in humans yet, and even in chickens, the bacterium appears to behave more like a commensal [23]. Therefore, it is less likely that *C. gallinacea* induces disease in humans.

Despite certain limitations to this study, such as a limited sample size and spread, this study provides clear evidence for the presence of all three avian *Chlamydia* species in both commercial and domestic chickens. While only one zoonotic event was observed in the commercial chicken sector, the transmission of *C. psittaci*, *C. gallinacea*, and *C. abortus* to backyard chicken owners seemed highly likely. Three people testing positive for *C. psittaci* and/or *C. gallinacea* experienced clinical signs suggestive of a *Chlamydia* infection. Although no significant correlation was found between infection and clinical signs, this may suggest a potential for *C. gallinacea* to induce disease in humans. Given the potential for severe disease in humans when infected with *C. psittaci* and *C. abortus*, immunosuppressed individuals should be cautious when caring for these animals. Moreover, as *C. abortus* is the enzootic agent of abortion in small ruminants and has been associated with abortion in humans, further research is needed to assess the risks posed to pregnant women caring for domestic chickens.

## Figures and Tables

**Table 1:** Descriptives and PCR results of commercial chicken samples. Prevalence rates are reported as “Number of positive chickens/number of tested chickens (% of positive chickens)” or as “Number of positive flocks/number of tested flocks (% of positive flocks)”. Cp = *C. psittaci*; Ca = *C. abortus*; Cg = *C. gallinacea*.

Sampling Location	Chicken Type	Age of Chickens at Sampling	Flock Size	Presence of Other Farm Animals	*C. psittaci* Prevalence	*C. gallinacea* Prevalence	*C. abortus* Prevalence	*Chlamydia* Prevalence	Mixed Infections
Farm 1	Broilers	35–42 days	20,000	Pigs	1/10 (10.0%)	0/10 (0.0%)	0/10 (0.0%)	1/10 (10.0%)	
Farm 2	Broilers	35–42 days	35,000	None	0/10 (0.0%)	3/10 (30.0%)	0/10 (0.0%)	3/10 (30.0%)	
Farm 3	Broilers	35–42 days	45,000	Dairy cattle	0/10 (0.0%)	0/10 (0.0%)	0/10 (0.0%)	0/10 (0.0%)	
Farm 4	Slow-growing broilers	35–42 days	10,000	None	0/10 (0.0%)	0/10 (0.0%)	0/10 (0.0%)	0/10 (0.0%)	
Farm 5	Layer hens	45 weeks	20,000	None	3/10 (30.0%)	0/10 (0.0%)	1/10 (10.0%)	3/10 (30.0%)	Cp/Ca
Farm 6	Broilers	35–42 days	45,000	None	6/10 (60.0%)	0/10 (0.0%)	0/10 (0.0%)	6/10 (60.0%)	
Farm 7	Broilers	35–42 days	30,000	Pigs	3/10 (30.0%)	0/10 (0.0%)	0/10 (0.0%)	3/10 (30.0%)	
Farm 8	Broilers	35–42 days	25,000	None	3/10 (30.0%)	1/10 (10.0%)	0/10 (0.0%)	4/10 (40.0%)	
Farm 9	Broilers	35–42 days	35,000	None	1/10 (10.0%)	1/10 (10.0%)	0/10 (0.0%)	1/10 (10.0%)	Cp/Cg
Farm 10	Broilers	35–42 days	45,000	None	4/10 (40.0%)	1/10 (10.0%)	0/10 (0.0%)	5/10 (50.0%)	
Farm 11	Broilers	35–42 days	20,000	None	5/10 (50.0%)	1/10 (10.0%)	1/10 (10.0%)	6/10 (60.0%)	Cp/Cg
Farm 12	Broilers	35–42 days	40,000	None	2/10 (20.0%)	0/10 (0.0%)	5/10 (50.0%)	6/10 (60.0%)	Cp/Ca
Farm 13	Broilers	35–42 days	35,000	None	1/10 (10.0%)	3/10 (30.0%)	2/10 (20.0%)	6/10 (60.0%)	
Farm 14	Layer hens	65 weeks	30,000	None	1/10 (10.0%)	1/10 (10.0%)	3/10 (30.0%)	4/10 (40.0%)	Ca/Cg
Farm 15	Broilers	35–42 days	25,000	Sheep, donkeys	0/10 (0.0%)	2/10 (20.0%)	0/10 (0.0%)	2/10 (20.0%)	
Farm 16	Unknown	Unknown	Unknown	Unknown	0/10 (0.0%)	2/10 (20.0%)	2/10 (20.0%)	4/10 (40.0%)	
Farm 17	Broilers	35–42 days	25,000	None	0/10 (0.0%)	1/10 (10.0%)	2/10 (20.0%)	2/10 (20.0%)	Ca/Cg
Farm 18	Broilers	35–42 days	30,000	None	0/10 (0.0%)	5/10 (50.0%)	7/10 (70.0%)	7/10 (70.0%)	Ca/Cg
Farm 19	Broilers	35–42 days	45,000	None	2/10 (20.0%)	1/10 (10.0%)	2/10 (20.0%)	2/10 (20.0%)	Cp/Ca and Cp/Ca/Cg
Farm 20	Broilers	35–42 days	30,000	Beef cattle	0/10 (0.0%)	0/10 (0.0%)	1/10 (10.0%)	1/10 (10.0%)	
**Prevalence in farm chickens**					32/200 (15.0%)	22/200 (11.0%)	26/200 (13.0%)	66/200 (33.0%)	
**Prevalence in farm flocks**					12/20 (60.0%)	12/20 (60.0%)	10/20 (50.0%)	18/20 (90.0%)	
Abattoir 1	Broilers	42 days	Unknown	Unknown	1/40 (2.5%)	2/40 (5.0%)	1/40 (2.5%)	3/40 (7.5%)	Ca/Cg
Abattoir 2	Layer hens	±2 years	17,000	None	2/40 (5.0%)	1/40 (2.5%)	0/40 (0.0%)	3/40 (7.5%)	
Abattoir 3	Layer hens	±2 years	25,500	Rabbits	6/40 (15.0%)	6/40 (15.0%)	4/40 (10.0%)	13/40 (32.5%)	Ca/Cg and Cp/Ca and Cp/Cg
Abattoir 4	Layer hens	±2 years	20,000	None	0/40 (0.0%)	1/40 (2.5%)	4/40 (10.0%)	4/40 (10.0%)	Ca/Cg
Abattoir 5	Layer hens	±2 years	40,500	None	3/40 (7.5%)	0/40 (0.0%)	1/40 (2.5%)	4/40 (10.0%)	
**Prevalence in abattoir chickens **					12/200 (6.0%)	10/200 (5.0%)	10/200 (5.0%)	27/200 (13.5%)	
**Prevalence in abattoir flocks**					4/5 (80.0%)	4/5 (80.0%)	4/5 (80.0%)	5/5 (100.0%)	
**Total prevalence in commercial chickens**					**44/400 (11.0%)**	**32/400 (8.0%)**	**36/400 (9.0%)**	**93/400 (23.3%)**	
**Total prevalence in commercial chicken flocks**					**16/25 (64.0%)**	**16/25 (64.0%)**	**14/25 (56.0%)**	**23/25 (92.0%)**	

**Table 2:** PCR analysis results of backyard chickens. Prevalence rates are reported as “Number of positive chickens/number of tested chickens (% of positive chickens)” or as “Number of positive locations/number of tested locations (% of positive locations)”.

	*C. psittaci* Prevalence	*C. gallinacea* Prevalence	*C. abortus* Prevalence	*Chlamydia* Prevalence
Positive locations	1/38 (2.6%)	9/38 (23.7%)	1/38 (2.6%)	10/38 (26.3%)
Positive chickens	1/76 (1.3%)	11/76 (14.5%)	2/76 (2.6%)	13/76 (17.1%)

**Table 3:** PCR results of human pharyngeal swabs. Prevalence rates are reported as “Number of positive humans/Number of tested humans (% of positive humans).

	*C. psittaci* Prevalence	*C. gallinacea* Prevalence	*C. abortus* Prevalence	*Chlamydia* Prevalence
Domestic chicken owners	3/54 (5.6%)	3/54 (5.6%)	1/54 (1.9%)	6/54 (11.1%)
Chicken farm employees	0/15 (0.0%)	0/15 (0.0%)	0/15 (0.0%)	0/15 (0.0%)
Abattoir workers	1/33 (3.0%)	0/33 (0.0%)	0/33 (0.0%)	1/33 (3.0%)
**Total**	**4/102 (3.9%)**	**3/102 (2.9%)**	**1/102 (1.0%)**	**7/102 (6.9%)**

**Table 4:** Descriptives of *Chlamydia*-positive humans; Cp = *C. psittaci*; Ca = *C. abortus*; Cg = *C. gallinacea*.

Human	Age	Gender	Contact with Other Birds	Contact with Other Animals	Clinical Signs	*Chlamydia* in Human	*Chlamydia* in Chickens
1	50	M	None	Daily with cat, dog, and horses	None	Cg	Cg
2	42	F	None	Daily with cat/dog and weekly with horses	Headache, stomachache, and dry skin	Cg	Cg
3	46	M	None	Daily with cat and weekly with cattle, sheep, and goats	Rhinitis and dry eyes	Cp and Cg	Cg
4	60	F	None	Daily with dog and sheep	None	Cp	None
5	36	M	None	None	COVID-19 infection with respiratory signs	Cp	None
6	55	M	None	None	None	Cp	No own chickens
7	21	M	Daily with pigeons, ducks, and geese	Daily with cat, dog, and pigs	None	Ca	Ca

## Data Availability

Data are contained within the article.
